# Accuracy of measuring half‐ and quarter‐value layers and appropriate aperture width of a convenient method using a lead‐covered case in X‐ray computed tomography

**DOI:** 10.1120/jacmp.v15i1.4602

**Published:** 2014-01-06

**Authors:** Kosuke Matsubara, Katsuhiro Ichikawa, Yuichi Murasaki, Ayaka Hirosawa, Kichiro Koshida

**Affiliations:** ^1^ Department of Quantum Medical Technology, Faculty of Health Sciences Institute of Medical, Pharmaceutical and Health Sciences, Kanazawa University Kanazawa Japan; ^2^ Department of Quantum Medical Technology, Graduate Course of Medical Science and Technology Kanazawa University Graduate School of Medicine Kanazawa Japan

**Keywords:** computed tomography, half‐value layer, quarter‐value layer, radiation dosage

## Abstract

Determination of the half‐value layer (HVL) and quarter‐value layer (QVL) values is not an easy task in X‐ray computed tomography (CT), because a nonrotating X‐ray tube must be used, which requires the assistance of service engineers. Therefore, in this study, we determined the accuracy of the lead‐covered case method, which uses X‐rays from a rotating X‐ray tube, for measuring the HVL and QVL in CT. The lead‐covered case was manufactured from polystyrene foam and a 4 mm thick lead plate. The ionizing chamber was placed in the center of the case and aluminum filters were placed 15 cm above the aperture surface. Aperture widths of 1.0, 2.0, and 3.0 cm for a tube voltage of 110 kV and an aperture width of 2.0 cm for the tube voltages of 80 and 130 kV were used to measure exposure doses. The results of the HVL and QVL were compared with those of the conventional nonrotating method. A 2.0 cm aperture was believed to be adequate, because of its small differences in the HVL and QVL in the nonrotating method and its reasonable exposure dose level. When the 2.0 cm aperture was used, the lead‐covered case method demonstrated slightly larger HVLs and QVLs (0.03‐0.06 mm for the HVL and 0.2‐0.4 mm for the QVL) at all the tube voltage settings. However, the differences in the effective energy were 0.1‐0.3 keV; therefore, it could be negligible in an organ‐absorbed dose evaluation and a quality assurance test for CT.

PACS numbers: 87.57.‐s; 87.57.Q‐; 87.57.uq

## I. INTRODUCTION

X‐rays used in radiographic systems are polyenergetic bremsstrahlung, and the type and total amount of filtration, together with the type of rectification, are the major factors affecting beam quality. For standard quality assurance testing and radiation dose reduction in patients, the half‐value layer (HVL) and quarter‐value layer (QVL) are often used as practical values to assess X‐ray beam quality.

To assess the beam quality of X‐rays used in radiographic systems, the HVL and QVL are measured for X‐ray beams in fluoroscopy, radiography, mammography, and computed tomography (CT). However, in CT, determining the HVL and QVL is not an easy task, because they should be measured with a nonrotating exposure mode, which requires the assistance of service engineers. Several techniques that do not require the nonrotating mode for measuring the HVL have been reported.^1^, ^2^, ^3^ Kruger et al.^(1)^ evaluated ring and localization techniques and found that they were accurate and reproducible at the isocenter. However, the ring technique has the disadvantage of requiring many high‐purity concentric aluminum rings, and the localization technique requires an ionization chamber support mechanism other than the patient table and some method to cantilever the chamber into the scan plane. Maia and Caldas^(2)^ developed tandem systems that were formed by a pencil ionization chamber with and without a specific covering, and they showed that the systems could possibly be used to measure the HVLs instead of the conventional method in CT. Although their systems could measure the HVLs while in a rotating exposure mode in CT, they would require chamber‐specific covering forms, which are difficult or time‐consuming to prepare.

In previous literature, another method that also does not require the nonrotating exposure mode for measuring the HVL has been described.^(3)^ This method uses a lead‐covered polystyrene foam case in the center of which an ionization chamber is placed. This lead‐covered case method is convenient, because the lead‐covered case could be easily manufactured in‐house and does not need to have a chamber‐ or scanner‐specific size and shape. However, the accuracy of this method has not been investigated until now. Therefore, in this study, we determined the accuracy of the lead‐covered case method for measuring the HVL and QVL in CT by comparing this method with the conventional “gold standard” method that uses the nonrotating exposure mode (the nonrotating method).

## II. MATERIALS AND METHODS

### A. CT system and dosimeter

A 16‐channel multidetector row CT scanner (SOMATOM Emotion; Siemens Healthcare, Erlangen, Germany) was used. A 6 cm^3^ general purpose ionization chamber (model 20X6‐6; Radcal, Monrovia, CA) and an electrometer (model 2026C; Radcal) were used to measure the exposure doses.

### B. Measurements of HVL and QVL

#### B.1 Lead‐covered case method

**Figure 1:**
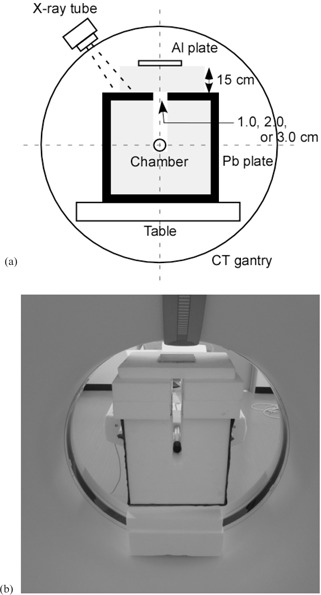
Experimental setup for the lead‐covered case method. The case was manufactured from polystyrene foam and covered with 4 mm thick lead plates. The size of the case was 30×30×15cm. Photograph of the experimental setup (a) and projection view (b) of the experimental setup.

We used a lead‐covered polystyrene foam case in the center of which an ionization chamber was placed. Figure 1 shows the experimental setup for the lead‐covered case method. We used a 4 mm thick lead plate as the cover material. When the aperture located at the upper surface was completely covered by the same lead plate, the exposure dose was undetectable at a maximum tube voltage of 130 kV. Two 4 mm thick lead plates with sharp cutting edges were placed on the same side of the aperture, so that we could adjust the aperture width by sliding the two plates. The polystyrene foam around the X‐ray passage between the aperture and the ionization chamber was removed to provide clearance for the X‐ray passage. The table was moved away from the gantry, and the case was placed in the CT gantry with a cuboid‐shaped polystyrene foam base. The height of the base was adjusted so that the chamber, set in the center of the case, was located at the isocenter. Aluminum filters were placed 15 cm over the aperture to minimize the effect of scattered X‐rays from the aluminum plates. In this way, we investigated the accuracy of this method for the HVL and QVL measurements and determined the appropriate aperture width for accurate measurement.

Exposure doses were measured using the following scan conditions: the tube voltages of 80, 110, and 130 kV for a 2.0 cm aperture and 110 kV for 1.0 and 3.0 cm apertures; a tube current of 200 mA; 1.0 s per rotation; a detector configuration of 16×1.20mm, and a large focal spot size. The start tube angle was set to 6 o'clock direction by operating in the service engineering mode.

#### B.2 Nonrotating method

**Figure 2:**
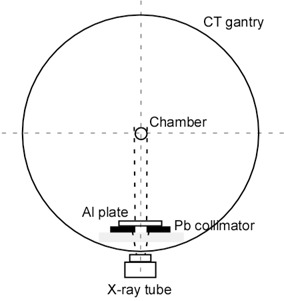
Projection view of the experimental setup for the nonrotating method. The X‐ray tube was positioned at the bottom of the scanner gantry and held stationary at that location. The ionization chamber was positioned free‐in‐air at the isocenter of the gantry.

Figure 2 shows the experimental setup for the nonrotating method. In the service engineering mode, the X‐ray tube was positioned at 6 o'clock direction, the ionization chamber was placed free‐in‐air at the isocenter of the gantry,^1^, ^4^ and the aluminum filters were placed on a 25 mm wide lead collimator 75 mm above the bottom surface of the gantry.

Exposure doses were measured using the following exposure conditions: the tube voltages of 80, 110, and 130 kV; a tube current of 50 mA; an exposure time of 1.0 s; a detector configuration of 16×1.20mm; and a large focal spot size.

#### B.3 Data collection for determining HVLs and QVLs

In this study, 10×10cm aluminum 1100 plates with a nominal thickness of 2.0 mm were used as attenuators. The thicknesses of the attenuators were measured using a digital caliper with an accuracy of 0.01 mm, and the thicknesses ranged from 1.97 to 2.00 mm.

After the initial exposure dose was measured without the attenuator, exposure measurements with attenuators were performed using the same parameters (i.e., adjusting the aluminum filters into the beam path until the resulting exposure was less than half the initial value to obtain the HVL and less than a quarter of the initial value to obtain the QVL). The exposure dose was measured repeatedly three times for each filter thickness, and the HVL and QVL were determined from the obtained mean exposure doses by the following procedure. First, a quartic (110 and 130 kV) or cubic (80 kV) approximation was derived from the relationship between the filter thickness and the obtained exposure dose. Second, the half‐ and quarter‐values of the initial exposure values were calculated from the approximation to determine the HVL and QVL. Third, effective energy was estimated from the HVL and the linear attenuation coefficient of aluminum.^(5)^

Homogeneity coefficients (HCs) that describe the polychromatic nature of the beam^(6)^ and quality indices (QIs) were calculated using Eqs. (1) and (2), as follows:

(1) $$HC=\frac{HVL}{QVL-HVL}$$

(2) $$QI=\frac{Effectiv\text{e e}nergy}{Tub\text{e v}oltage}$$

## III. RESULTS

### A. HVL and QVL at different aperture widths

**Figure 3:**
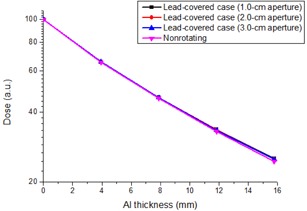
Attenuation curves for the aluminum filters among the different aperture widths of the lead covers.

The HVLs and QVLs obtained with the tube voltage of 110 kV at 1.0, 2.0, and 3.0 cm apertures by the lead‐covered case and nonrotation methods are shown in Fig. 3 and Table 1. The nonrotating method showed slightly smaller HVLs and QVLs than those obtained by the lead‐covered case method, whereas the latter method showed 0.03‐0.09 mm (0.31%‐1.2%) and 0.3‐0.4 mm (2.2%‐2.8%) larger HVLs and QVLs, respectively. The differences in the HVLs and QVLs were the smallest for aperture widths of 2.0 and 3.0 cm, respectively. The accuracy and reproducibility of the dose measurement were sufficient, because the coefficients of variance for the thrice repeated dose measurements for the respective filter thicknesses were <0.51% for all the measurements. As a result, the lead‐covered case method overestimated the effective energy by 0.1‐0.3 keV (0.18%‐0.73%), underestimated the HC by 0.014‐0.030 (1.8%‐3.8%), and overestimated the QI by 0.001‐0.003 (0.18%‐0.73%).

**Table 1:** Results for the half‐value layers (HVLs) and quarter‐value layers (QVLs) at different aperture widths

*Method*	*Aperture Width (cm)*	*HVL (mm)*	*QVL (mm)*	*Effective Energy (keV)*	*Homogeneity Coefficient*	*Quality Index*
Lead‐covered case	1.0	6.88	15.8	49.6	0.770	0.451
	2.0	6.87	15.8	49.6	0.773	0.451
	3.0	6.93	15.7	49.8	0.786	0.453
Nonrotating	N/A	6.84	15.4	49.5	0.800	0.450

N/A=not applicable.

### B. HVL and QVL at different tube voltage

The HVLs and QVLs obtained at 80, 110, and 130 kV by the two methods are shown in Table 2. In the lead‐covered case method, the aperture width was set to 2.0 cm. The nonrotating method showed slightly smaller HVLs and QVLs at all the tube voltage settings; the lead‐covered case method showed 0.03‐0.06 mm (0.31%‐1.3%) and 0.2‐0.4 mm (1.8%‐2.3%) larger HVLs and QVLs, respectively. The accuracy and reproducibility of the dose measurement were sufficient, because the coefficients of variance for the thrice‐repeated dose measurements for the respective filter thicknesses were <0.92% for all measurements. The lead‐covered case method overestimated the effective energy by 0.1‐0.3 keV (0.18%‐0.59%), underestimated the HC by 0.008‐0.027 (0.95%‐3.4%) and overestimated the OT by 0.001‐0.003 (0.18%‐0.59%).

**Table 2:** Results for the HVLs and QVLs at different tube voltage settings

*Tube Voltage (kV)*	*Method*	*HVL (mm)*	*QVL (mm)*	*Effective Energy (keV)*	*Homogeneity Coefficient*	*Quality Index*
80	Lead‐covered case	4.96	11.5	41.8	0.760	0.522
	Nonrotating	4.90	11.3	41.5	0.768	0.519
110	Lead‐covered case	6.87	15.8	49.6	0.773	0.451
	Nonrotating	6.84	15.4	49.5	0.800	0.450
130	Lead‐covered case	8.02	18.1	54.6	0.793	0.420
	Nonrotating	7.97	17.8	54.4	0.815	0.419

## IV. DISCUSSION

In this study, we determined the accuracy of the lead‐covered case method for measuring the HVL and QVL in CT. Although this method showed 0.03‐0.09 mm and 0.2‐0.4 mm larger HVLs and QVLs, respectively, compared with the conventional nonrotation method, this method overestimated the effective energy only by 0.1‐0.3 keV.

When organ‐absorbed doses are calculated, the mass energy coefficient ratio of each organ to air would be required. However, because the coefficient changes according to the photon energy,^7^, ^8^ the photon energy has to be estimated for calculating the organ‐absorbed dose. The effective energy of a polyenergetic X‐ray beam is defined as being equivalent to the energy of a monoenergetic X‐ray beam that has the same HVL. Therefore, estimating the effective energy of a polyenergetic X‐ray is necessary for calculating the organ‐absorbed dose.

The results of the HVL, QVL, HC, and QI for the 1.0, 2.0, and 3.0 cm apertures were similar. From these results, it was believed that the 1.0 cm aperture had an accuracy similar to that of the 2.0 cm aperture. However, when a 1.0 cm aperture was used, the exposure doses become relatively small, and the smaller dose levels may affect the dose measurement accuracy, depending on the CT system used. For the 3.0 cm aperture, although the HC and QVL values were the nearest to those of the nonrotating method, there were tendencies that the HVL was larger and the effective energy was overestimated, as compared with the other apertures. However, the coefficients of variance for the thrice‐repeated dose measurements for the respective filter thicknesses were <0.51% for all the measurements, and we believe the differences in the HVLs between 2.0 and 3.0 cm apertures were not significant. We also believe that these differences in the HVLs between the two methods (0.03‐0.09 mm) could be negligible in an organ‐absorbed dose evaluation and a quality assurance test for CT. To measure the HVL as accurately as possible by the lead‐covered case method, we suggest that the aperture width be adjusted to 2.0 cm.

The differences in the QVLs between the two methods were 0.3‐0.4 mm, which were larger than those of the HVLs. We speculated that the QVLs were much larger than those of the HVLs, because under the attenuation conditions of the QVL measurements, the exposure doses were relatively small (half of the HVL dose). In addition, the exposure time in the lead‐covered case method was much shorter than that of the nonrotating method, because the time during which the X‐rays could reach the chamber in the rotational exposure was limited. Therefore, the dose levels of the lead‐covered case method were significantly smaller than those of the nonrotating method. In this study, the initial exposure doses of the lead‐covered case method were 4.8%‐13% of those of the nonrotating method. Under such small dose conditions, bias in the dose measurements due to leakage currents and other factors was unavoidable; therefore, it was believed that the bias caused larger QVLs. However, estimating the QVL is generally not as routine as estimating the HVL, because it is necessary to characterize the polyenergetic nature of an X‐ray beam only for determining the HC.^(7)^ Accurate measurement of the QVL for determining HC may require the application of the nonrotating method.

The nonrotating method also showed smaller HVLs and QVLs at all the tube voltage settings (80, 110, and 130 kV) than those obtained by the lead‐covered case method at an aperture width of 2.0 cm. Although the HVLs in the lead‐covered case method were 0.03‐0.06 mm (0.31%‐1.3%) larger, we believe that the differences in the HVLs could also be negligible at all the tube voltage settings.

The methods using concentric aluminum rings or localizer radiographs proposed by Kruger et al.^(1)^ had 0.69% and 0.97% errors in the estimation of the HVLs, respectively, which were almost equivalent to those in our study. The lead‐covered case method has the advantage that the case could be easily manufactured in‐house from lead plates and polystyrene foam. We expect that anyone who wants to measure the HVL could manufacture the lead‐covered case, and the difference in the HVLs among the measurements should be extremely negligible. However, the lead‐covered case method has the following two disadvantages: i) exposure doses are relatively small and may cause larger HVL; and ii) exposure values are unstable because the tube start angle cannot be adjusted to a certain position unless the service engineering mode is used. Therefore, we strongly recommend that a high tube current and long rotation time be used, that a chamber be put closer to the isocenter (the difference caused by the attenuation of air should not be significant), and that measurements be repeated as many times as possible when the lead‐covered case method is applied.

Our study had some limitations. First, we used only one type of CT scanner. Although we believe that similar results will be obtained with other types of CT scanners, such studies with other CT scanners should be performed to determine the accuracy of the lead‐covered case method for measuring the HVL and QVL in CT in more detail. Second, we could not use a pencil ionization chamber, which is generally used for CT measurements in this study. We believe that there is no need to use a pencil ionization chamber instead of the general‐purpose ionization chamber; however, it may be possible that the obtained HVLs and QVLs will differ depending on the chamber used. Third, we did not evaluate the size of the case. It is possible that a large amount of scattered radiation would be measured in the chamber if the size was too small, and it is difficult or impossible to manufacture, carry, set the case into the gantry, and place the absorbers on the gap if the size is too large. Because adding a heavy and sizeable lead‐covered case to the already over‐burdened CT test equipment ensemble would seem to make it unusable for general physics testing, the appropriate size of the lead‐covered case needs to be investigated. Finally, although the polystyrene foam around the X‐ray passage between the aperture and the ionization chamber was removed to provide clearance for the X‐ray passage, the scatter from the polystyrene form might contribute to the deviation from the lead‐covered case method. Therefore, the effect of scatter from the polystyrene form should be investigated to improve the accuracy of this method.

## IV. CONCLUSIONS

The HVLs and QVLs were 0.03‐0.06 mm and 0.2‐0.4 mm larger, respectively, in the lead‐covered case method than those in the conventional nonrotating method. The corresponding errors in effective energy were 0.1‐0.3 keV This finding indicated that the lead‐covered case method could give reasonable accuracy for the HVL and QVL measurement. The appropriate aperture width was found to be 2.0 cm.

## ACKNOWLEDGMENTS

We would like to thank Dr. Shuji Koyama and Dr. Takanori Hara for advice and discussion regarding the lead‐covered case method, and Siemens Japan K.K. and Marubun Tsusho for operating the service engineering mode.
